# Normal ranges of non-invasive left ventricular myocardial work indices in healthy young people

**DOI:** 10.3389/fped.2022.1000556

**Published:** 2022-09-09

**Authors:** Xiuxia Luo, Quanrong Ge, Jin Su, Ning Zhou, Ping Li, Xu Xiao, Yan Chen, Dong Wang, Yujing Ma, Li Ma, Yongsheng Zhu

**Affiliations:** Department of Ultrasonography, Shenzhen Hospital, Southern Medical University, Shenzhen, China

**Keywords:** young people, echocardiography, myocardial work, two-dimensional speckle tracking imaging, peak strain dispersion, myocardial strain

## Abstract

**Objectives:**

Echocardiographic global myocardial work (GMW) indices recently emerged to non-invasively evaluate left ventricular (LV) myocardial performance with less load-dependence than LV ejection fraction (LVEF) or global longitudinal strain (GLS). Yet, few data exist on the descriptions of LV GMW indices in young people. We therefore aimed to provide normal reference values of LV GMW in a healthy young cohort, and simultaneously to investigate factors associated with non-invasive GMW indices.

**Materials and methods:**

A total of 155 healthy young people (age 10–24 years, 59% male) underwent transthoracic echocardiography were recruited and further stratified for age groups and divided by gender. Two-dimensional speckle-tracking echocardiography (2D-STE) were performed to determine LV GLS, peak strain dispersion (PSD) and GMW indices, which include global work index (GWI), global constructive work (GCW), global wasted work (GWW), and global work efficiency (GWE). LV peak systolic pressure was assumed to be equal to the systolic brachial artery cuff blood pressure.

**Results:**

Age and gender specific normal ranges for LV GMW indices were presented. On multivariable analysis, GWI and GCW correlated more closely with systolic blood pressure (SBP) than LV GLS, while both GWW and GWE independently correlated with PSD (*P* < 0.05 for all). There were no associations between any of the GMW indices with age, sex, body mass index, heart rate, left ventricular mass index as well as LV sizes or LVEF. Of noted, LV GMW indices had good intra-observer and inter-observer reproducibility.

**Conclusion:**

We reported echocardiographic reference ranges for non-invasive LV GMW indices in a large group of healthy young subjects, which are reproducible and reliable, and thus can be further used when assessing subclinical dysfunction in young people with myocardial diseases.

## Introduction

Accurate detection of subtle left ventricular (LV) contractile dysfunction may be of clinical importance in young patients with various heart diseases to avoid irreversible consequent LV deterioration in later life. The recently employed echocardiographic surrogates of LV contractility, such as LV ejection fraction (LVEF), tissue Doppler imaging (TDI), and most recently myocardial strain derived from speckle-tracking echocardiography (STE), are load-dependent and susceptible to misinterpretation of the true myocardial contractility as they are all markedly influenced by afterload (1–3).

In recent years, non-invasive assessment of LV myocardial work (LVMW), a new echocardiographic tool based on STE-derived longitudinal strain and peripheral systolic blood pressure (SBP) (as a surrogate of LV systolic pressure), has attracted widespread attention (4). It is thought to be less load-dependent compared to LVEF or global longitudinal strain (GLS), integrating information on LV active systolic and diastolic work, thus provides more comprehensive quantification of LV myocardial contractility. Importantly, the application of echocardiographic LVMW has been validated against invasive work measurements and reflected cardiac metabolism (5–7). This novel method offers serial information of LV function involving constructive work, wasted work, and energy consumption, thus adding insights into both cardiac mechanics and the pathophysiology of cardiovascular disease states.

With the growing interest in non-invasive LVMW, the knowledge of normal reference values specific to young population are requisite. Despite robust normative data for the performance of myocardial work characteristics are well-established in adult populations (8–11), data in young people to date are still insufficient and based on relatively small cohorts (12–15). Therefore, in this study we aimed (1) to determine the reference values for echocardiography-derived LV global myocardial work (GMW) in a large cohort of healthy young people, and (2) to further investigate the influence of age, sex, and other clinical factors on GMW indices.

## Materials and methods

### Study population

From May 2019 to July 2021, a total of 165 healthy young people aged 10–24 years according to the World Health Organization (WHO) definition were prospectively enrolled in our study (16). Healthy subjects were determined by absence of cardiovascular risk factors and structural or functional abnormalities during the echocardiogram, normal physical cardiac examination, and electrocardiogram. The exclusion criterion was the presence of suboptimal echocardiographic images or cardiac rhythm abnormalities such as ventricular and supraventricular arrhythmia. After excluding ten subjects with poor image quality, 155 volunteers with adequate image quality remained for study, stratified for sex. Meanwhile, the studied population were further categorized into three age groups: 10–14 years, 15–19 years, and 20–24 years (17). This study has been approved by the Medical Ethics Committee of Shenzhen Hospital of Southern Medical University, with a waiver of the requirement for informed consent given the retrospective nature of the research.

### Blood pressure measurement

Blood pressure was obtained by measuring the average value of the three consecutive measurements in the sitting position, at least 1 min apart and after 5 min of rest, using the Datascope Accutorr Plus (Datascope Corporation, Mahwah, NJ, United States), which has been validated for use in local Chinese adolescents (18).

### Conventional echocardiography

Comprehensive transthoracic echocardiography was performed with the volunteer in the left lateral position employing breath holds, using a Vivid E9 or E95 system (GE Healthcare, Horten, Norway). Two-dimensional (2D) images from the standard three LV apical views (four-chamber, two-chamber, and long-axis) were acquired during three consecutive cycles, with an image frame rate of 50–70 frames per second. All echocardiographic images were digitally stored for offline analysis with the dedicated software (EchoPAC version 203, GE Healthcare, Horten, Norway). Parasternal, apical, and subcostal views were used to acquire 2D, color, pulsed-wave, and continuous wave Doppler data according to current recommendations (19). LV volumes and LVEF were estimated using biplane Simpson method. Left ventricular mass index (LVMI) was obtained according to the corrected method of the American Society of Echocardiography (ASE) and indexed by height raised to a power of 2.7 (20). Left atrial maximal volume (LAV) measured from apical four−chamber and two−chamber views (biplane Simpson) was indexed for body surface area (BSA) to determine maximal LA volume index (LAVi). The peak early (E) and late (A) diastolic velocities were calculated on transmitral flow pulsed-wave recordings. Also, early diastolic septal mitral annular velocity (e′) was obtained from pulse wave velocity of spectral tissue Doppler imaging and E/e′ ratio was then calculated (19).

### Left ventricular global myocardial work analysis

**FIGURE 1:**
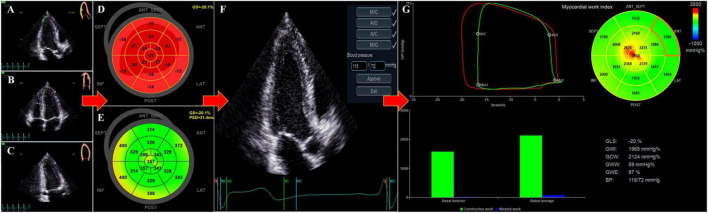
Non-invasive determination of left ventricular (LV) myocardial work from echocardiography. Using automated function imaging (AFI) software, images of the apical long–axis **(A)**, apical four– **(B)** and two–chamber **(C)** views were analyzed to determine LV global longitudinal strain (GLS) **(D)** and peak strain dispersion (PSD) **(E)**. After adjusting the event timing of aortic and mitral valve closure and opening in the apical long-axis view and inputting the blood pressure (BP) value **(F)**, the Bull’s-eye images of 17 LV segments for myocardial work index or myocardial work efficiency were presented with global myocardial work values **(G)**, including global work index (GWI), global constructive work (GCW), global wasted work (GWW), and global work efficiency (GWE).

Quantification of LV GMW indices were performed offline using proprietary software package (Automated Functional Imaging; EchoPAC version 203, GE Healthcare), which integrates LV strain measurements with blood pressure recordings (5). Peak LV systolic pressure is assumed to be equal to the systolic brachial artery cuff blood pressures, measured immediately prior to the echocardiographic study. Images of the apical four−chamber, apical two−chamber, and apical LV long−axis views were used to derive LV GLS and peak strain dispersion (PSD) (Figures 1A–E). PSD was calculated from the standard deviation of the time to peak longitudinal strain values of all 17 LV segments. The automated tracking of the LV endocardial contour provided by software could be verified in real time and corrected by manual to ensure the inclusion of the entire LV thickness in all observed echocardiographic views. Once GLS was determined, the timing of aortic and mitral valve closure and opening were confirmed by aligning the event time in the apical long-axis view (Figure 1F). We further provided the program with brachial artery blood pressure value, to facilitate the derivation of the following LVMW indices (Figure 1G):

1. Global work index (GWI): the total work within the area of the global LV pressure-strain loop calculated from mitral valve closure to opening (mmHg%).
2. Global constructive work (GCW): positive work performed by LV during systole that is productive, including both shortening of the muscle during systole and lengthening of the muscle during isovolumic relaxation (IVR) (mmHg%).
3. Global wasted work (GWW): negative work performed by LV during systole that is not productive, including both lengthening of the muscle during systole and shortening of muscle in IVR (mmHg%).
4. Global work efficiency (GWE): GCW/(GCW + GWW) (0–100%).

### Reproducibility analysis

Intra-observer and inter-observer variabilities of LV GMW data were evaluated in 20 random subjects by means of intraclass correlation coefficients (ICCs). For the inter-observer variability, the differences between two independent sonographers who were blinded to the results of the other were evaluated. For the intra-observer variability, the differences between two observations made by the same sonographer after 2 weeks to avoid recall bias were also assessed. The criteria for ICCs were: “excellent” if > 0.74, “good” if 0.60–0.74, “fair” if 0.40–0.59, and “poor” if < 0.40 (12, 21).

### Statistical analysis

Collected data were computerized and analyzed using SPSS 22.0 (IBM SPSS, Statistics, Chicago, IL, United States) statistical software. Data normality was tested using Shapiro-Wilk method. Continuous variables are expressed as mean ± standard deviation (SD). Differences in continuous variables between two groups were analyzed using independent sample *t*-test or the Mann-Whitney *U* test as appropriate, while comparisons between three groups were made with a one-way analysis of variance (ANOVA) with Tukey’s *post-hoc* or Kruskal-Wallis test, as appropriate. The association of GMW indices with demographics and echocardiographic measures was performed using Pearson’s or Spearman’s correlation coefficient as appropriate. Further univariable linear regression and multivariable linear regression analyses were performed to determine the independent correlates between GMW indices and standard and advanced echocardiographic parameters. For multiple linear regression models, multicollinearity was also examined by computation of variance inflation factor. In case that collinear variables existed, the variable with the highest correlation coefficient was included. All *P*-values were reported as two-tailed, with < 0.05 considered as statistical significance.

## Results

### Demographic and conventional echocardiographic characteristics

The quantification of LV GLS, PSD as well as GMW by 2D-STE software was feasible in 155 out of 165 (93.9%) participants (17 ± 3 years, 92 males), with 10 individuals excluded for poor acoustic window. Demographic characteristics and conventional echocardiographic information were presented by gender in Table 1. There was no significant gender difference for age, body mass index (BMI), and heart rate (HR) (*P* > 0.05 for all). Height, weight, BSA as well as brachial cuff SBP were significantly higher in young males compared with females. Of noted, SBP in the youngest group of 10–14 years was significantly lower than that in the other two groups (*P* < 0.05 for both), while no significant difference in SBP was shown between the two older groups of young subjects (Table 2).

**TABLE 1:** Demographic and conventional echocardiographic characteristics in heathy young people between genders.

Variables	Total (*n* = 155)	Male (*n* = 92)	Female (*n* = 63)	*P*-value
Ages, years	17.1 ± 3.0	17.2 ± 2.9	16.9 ± 3.2	0.472
Height, cm	165 ± 10	169 ± 8	158 ± 7	<0.001
Weight, kg	57 ± 14	61 ± 14	53 ± 12	<0.001
BSA, m^2^	1.6 ± 0.2	1.7 ± 0.2	1.5 ± 0.2	<0.001
BMI, kg/m^2^	20.9 ± 3.9	21.0 ± 3.9	20.9 ± 3.9	0.651
Heart rate, bpm	65 ± 9	65 ± 10	66 ± 8	0.290
SBP, mmHg	112 ± 10	115 ± 9	109 ± 10	<0.001
DBP, mmHg	65 ± 8	66 ± 8	65 ± 8	0.441
LVIDd, mm	46 ± 4	47 ± 4	43 ± 4	<0.001
LVIDs, mm	30 ± 4	31 ± 3	28 ± 3	<0.001
IVSd, mm	7.1 ± 1.3	7.6 ± 1.3	6.3 ± 1.0	<0.001
LVPWd, mm	6.8 ± 1.1	7.2 ± 1.0	6.2 ± 0.9	<0.001
LVM/height^2.7^, g/m^2.7^	25.3 ± 6.3	27.2 ± 6.3	22.6 ± 5.1	<0.001
LVEDV, ml	94 ± 21	103 ± 20	80 ± 14	<0.001
LVESV, ml	34 ± 10	38 ± 9	28 ± 7	<0.001
LVEF, %	64 ± 4	63 ± 4	66 ± 5	0.001
LAV, ml	30.7 ± 8.9	32.7 ± 8.8	27.9 ± 8.3	0.001
LAVi, ml/m^2^	18.9 ± 4.4	19.3 ± 4.3	18.3 ± 4.6	0.156
E/A	2.4 ± 0.7	2.4 ± 0.7	2.3 ± 0.7	0.749
e′, cm/s	13 ± 2	13 ± 2	13 ± 2	0.866
E/e′	7.0 ± 1.6	7.0 ± 1.5	7.1 ± 1.6	0.611

Data are presented as mean ± SD. BSA, body surface area; BMI, body mass index; SBP, systolic blood pressure; DBP, diastolic blood pressure; LVIDd, left ventricular internal dimension at end-diastole; LVIDs, left ventricular internal dimension at end-systole; IVSd, interventricular septal diameter; LVPWd, left ventricular posterior wall diameter; LVM, left ventricular mass; LVEDV, left ventricular end-diastolic volume; LVESV, left ventricular end-systolic volume; LVEF, left ventricular ejection fraction; LAV, maximal left atrial volume; LAVi, maximal left atrial volume indexed to BSA; E, early mitral inflow velocity; A, late mitral inflow velocity; e′, peak early diastolic velocity of the septal mitral annulus (tissue Doppler image).

**TABLE 2:** Brachial blood pressure and advanced echocardiographic parameters by age groups.

Variables	10–14 years (*n* = 40)	15–19 years (*n* = 79)	20–24 years (*n* = 36)	*P*-value
SBP, mmHg	109 ± 10	113 ± 9*	115 ± 9*	0.008
DBP, mmHg	63 ± 8	65 ± 8	69 ± 7*	0.002
LV GLS, %	–20.0 ± 1.8	–19.0 ± 1.5*	–19.0 ± 1.2*	0.006
GWI, mmHg%	1,767 ± 195	1,715 ± 180	1,738 ± 172	0.302
GCW, mmHg%	1,920 ± 197	1,889 ± 180	1,911 ± 172	0.604
GWW, mmHg%	59 ± 21	64 ± 27	69 ± 22	0.289
GWE, %	96.5 ± 1.2	96.2 ± 1.4	96.0 ± 1.3	0.289

Data are presented as mean ± SD. SBP, systolic blood pressure; DBP, diastolic blood pressure; LV GLS, left ventricular global longitudinal strain; GWI, global work index; GCW, global constructive work; GWW, global wasted work; GWE, global work efficiency. *P < 0.05 compared with the age group of 10–14 years.

As expected, the young males had significantly larger LVMI, wall thickness, LAV, LV dimensions and volumes with lower LVEF when compared to female subjects (*P* ≤ 0.001 for all). There were no statistically significant differences in LAVi, mitral E/A ratio, mitral annulus e′ and E/e′ ratio between gender (all *P* > 0.05).

### 2D strain measurements and global myocardial work indices

Normal LV GMW parameters as well as 2D Strain measurements consisted of LV GLS and PSD were detailed by gender in Table 3. No significant differences were found in PSD and any component of GMW indices between sexes (*P* > 0.05 for all). However, the absolute value of LV GLS was slightly lower in young male (*P* = 0.001). Furthermore, the absolute value of LV GLS in the youngest group of 10–14 years was slightly higher than that in the other two groups (*P* < 0.05 for both), while no significant difference in GLS was shown between the two older groups of young subjects (Table 2). Of noted, there were no significant differences in GMW indices across age group as displayed in Table 2.

**TABLE 3:** Advanced echocardiographic characteristics in heathy young people between genders.

Variables	Total (*n* = 155)	Male (*n* = 92)	Female (*n* = 63)	*P*-value
LV GLS, %	–19.3 ± 1.6	–18.9 ± 1.4	–19.9 ± 1.6	0.001
PSD, ms	34.1 ± 5.0	34.1 ± 4.9	34.0 ± 5.1	0.890
GWI, mmHg%	1,734 ± 182	1,721 ± 170	1,752 ± 199	0.463
GCW, mmHg%	1,902 ± 182	1,893 ± 164	1,917 ± 206	0.543
GWW, mmHg%	64 ± 25	66 ± 24	60 ± 25	0.080
GWE, %	96.2 ± 1.3	96.1 ± 1.3	96.3 ± 1.3	0.140

Data are presented as mean ± SD. LV GLS, left ventricular global longitudinal strain; PSD, peak strain dispersion; GWI, global work index; GCW, global constructive work; GWW, global wasted work; GWE, global work efficiency.

### Correlations between global myocardial work indices and other parameters

**FIGURE 2:**
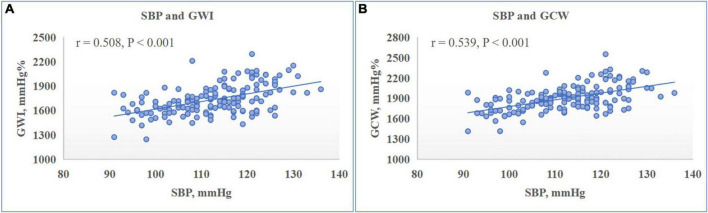
Relationship between left ventricular (LV) myocardial work indices and systolic blood pressure (SBP). Scatter plot presented the significantly positive correlation between LV global work index (GWI) **(A)** and global constructive work (GCW) **(B)** with SBP.

**FIGURE 3:**
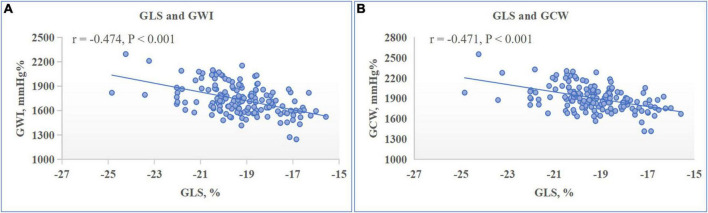
Relationship between left ventricular (LV) myocardial work indices and LV global longitudinal strain (GLS). Scatter plot presented the significantly negative correlation between LV global work index (GWI) **(A)** and global constructive work (GCW) **(B)** with LV GLS.

**FIGURE 4:**
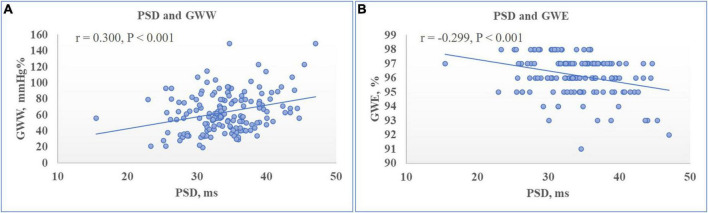
Relationship between left ventricular (LV) myocardial work indices and LV peak strain dispersion (PSD). Scatter plot showed that PSD was positively correlated to LV global wasted work (GWW) **(A)** while negatively correlated to global work efficiency (GWE) **(B)**.

There were no significant associations between any of LV GMW indices with sex, BMI, HR, mitral annulus e′ or LV dimensions and volumes. On both univariate and multivariate analysis as detailed in Table 4, both GWI and GCW presented significant correlations with SBP (standardized β-coefficient: 0.843 and 0.878 respectively, both *P* < 0.001) (Figure 2), LV GLS (standardized β-coefficient: –0.777 and –0.796 respectively, both *P* < 0.001) (Figure 3), and E/e′ (standardized β-coefficient = 0.148, *P* < 0.001; standardized β-coefficient = 0.099, *P* = 0.002, respectively). Moreover, both GWW and GWE significantly correlated with PSD (standardized β-coefficient = 0.250, *P* = 0.002; standardized β-coefficient = −0.208, *P* = 0.007, respectively) (Figure 4) and E/A (standardized β-coefficient = −0.165, *P* = 0.038; standardized β-coefficient = 0.162, *P* = 0.036, respectively). GWE was also found to be inversely correlated with LV GLS (standardized β-coefficient = −0.306, *P* < 0.001), while GWW was not associated with SBP and GLS. There were no associations between any of GMW indices with age, BSA, LVMI and LVEF on multivariate analysis. Noticeably, the β- standardized coefficients from multivariate linear regression model revealed that GWI and GCW were more closely correlated with SBP than LV GLS.

**TABLE 4:** Univariable and multivariable analysis for global myocardial work parameters.

	Univariable analysis		Multivariable analysis	
	Standardized coefficients	*P*-value	Standardized coefficients	*P*-value
**Global work index (mmHg%)**				
SBP	0.508	<0.001	0.843	<0.001
DBP	0.157	0.052		
LVM/height^2.7^	0.190	0.018		
E/e′	0.277	0.001	0.148	<0.001
LVEF	0.315	<0.001		
LV GLS	–0.474	<0.001	–0.777	<0.001
**Global constructive work (mmHg%)**				
SBP	0.539	<0.001	0.878	<0.001
DBP	0.192	0.017		
LVM/height^2.7^	0.138	0.086		
E/e′	0.225	0.005	0.099	0.002
LVEF	0.334	<0.001		
LV GLS	–0.471	<0.001	–0.796	<0.001
**Global wasted work (mmHg%)**				
Age	0.194	0.016		
BSA	0.167	0.037		
SBP	0.217	0.007		
DBP	0.242	0.002		
IVSd	0.173	0.031		
E/A	–0.227	0.005	–0.165	0.038
LV GLS	0.287	<0.001		
PSD	0.300	<0.001	0.250	0.002
**Global work efficiency (%)**				
Age	–0.169	0.036		
BSA	–0.170	0.034		
DBP	–0.196	0.015		
IVSd	–0.182	0.023		
E/A	0.224	0.005	0.162	0.036
LV GLS	–0.404	<0.001	–0.306	<0.001
PSD	–0.299	<0.001	–0.208	0.007

SBP, systolic blood pressure; DBP, diastolic blood pressure; LVM, left ventricular mass; BSA, body surface area; IVSd, interventricular septal diameter; LVEF, left ventricular ejection fraction; E, early mitral inflow velocity; A, late mitral inflow velocity; e′, peak early diastolic velocity of the septal mitral annulus (tissue Doppler image); LV GLS, left ventricular global longitudinal strain; PSD, peak strain dispersion.

### Repeatability and reproducibility

As it has been shown in Table 5, intra-observer and inter-observer analyses showed good repeatability and reproducibility in all LV GMW indices.

**TABLE 5:** Intra-observer and inter-observer variability.

	Intra-observer variability			Inter-observer variability		
	ICCs	95% CI	*P*-value	ICCs	95% CI	*P*-value
LV GLS	0.970	0.928–0.987	<0.001	0.947	0.874–0.978	<0.001
PSD	0.936	0.849–0.974	<0.001	0.893	0.740–0.956	<0.001
GWI	0.966	0.899–0.987	<0.001	0.941	0.857–0.975	<0.001
GCW	0.977	0.938–0.991	<0.001	0.953	0.875–0.981	<0.001
GWW	0.897	0.755–0.957	<0.001	0.817	0.557–0.924	<0.001
GWE	0.858	0.663–0.941	<0.001	0.780	0.464–0.909	0.001

ICCs, intraclass correlation coefficients; CI, confidence interval; LV GLS, left ventricular global longitudinal strain; PSD, peak strain dispersion; GWI, global work index; GCW, global constructive work; GWW, global wasted work; GWE, global work efficiency.

## Discussion

In present study, we demonstrated the normal echocardiographic reference values for LV GMW indices in a large cohort of healthy young people. Also, we found no influence of age, sex, BMI, HR, mitral annulus e′, LVMI, LV sizes or LVEF on GMW indices. Furthermore, our finding disclosed that both GWI and GCW were more closely correlated with SBP than LV GLS, while both GWW and GWE were independently correlated with PSD. Importantly, our study showed that non-invasive myocardial work analysis was feasible in young people with a favorable intra- and inter-observer variability, which agreed with previously reported series, thus enhancing the possibility of a promising application of LVMW in clinical practice.

To date, only three similar studies were conducted regarding the normal ranges of GMW in healthy young people (12, 13, 22). Specifically, the values of LV GMW indices including GWI, GCW, GWW, and GWE in Pham’s study (12) with 81 Asian healthy adolescents (age 10–21 years) were quite similar to our data. However, the other two studies showed slightly higher mean values of GWI and GCW in Western children and adolescents (13, 22). In addition, the mean GWW in our study was similar to the result (69 ± 46 mmHg%) from an international multi-center study in pediatric population (mean age of 10.6 ± 4.5 years, 61% males) (22), while lower than that in Tretter’s study (84 ± 28 mmHg%) with 52 healthy adolescents (age 11–19 years, 62% males) (13). The differences of LV GMW indices between studies maybe contributed to the ethnic difference and different levels of image quality. A recently published meta-analysis displayed normal mean values of GWI and GCW among the adult studies were 2,010 mmHg% (95% CI: 1,907–2,113 mmHg%) and 2,278 mmHg% (95% CI: 2,186–2,369 mmHg%), respectively. Mean GWW was 80 mmHg% (95% CI: 73–87 mmHg%), and mean GWE was 96.0% (95% CI: 96–96%). When compared to GMW indices in normal adults (8, 10, 11, 23), the mean values of GWI, GCW and GWW were lower in our young cohort, which may be associated with age-related endothelial dysfunction and arterial stiffness accompanied by increasing systolic blood pressure. Actually, the analyses from the Characteristics and Course of Heart Failure STAges A/B and Determinants of Progression (STAAB) cohort study depicted GMW indices were stable below the age of 45 years and then the values of GWI, GCW as well as GWW increased thereafter (10). Of noted, GWE remains relatively constant (average 96%) from adolescence into adulthood in healthy population (8–10, 12, 13, 22, 23). To the best of our knowledge, this is the largest study to provide normal echocardiographic values of non-invasive LV GMW indices among healthy young subjects, which may be incorporated into echocardiography laboratories to facilitate future assessment of myocardial performance in youth with congenital and acquired heart diseases.

It is still controversial whether gender contributed to variations in normal values of GMW. A growing body of literature has demonstrated no gender difference in GMW indices. Conversely, a recent meta-analysis of 12 studies, including nearly 1,665 adult subjects, indicated slight gender difference of GMW indices in healthy population (11). These incongruent findings might be due to the distribution of age, sex, and ethnicity in the different cohorts. In our study, LVEF and GLS as parameters of LV systolic function showed slightly more favorable values in female, which was similar to the findings of published studies (3, 10, 12, 19). Nevertheless, myocardial work measures that incorporate both intrinsic systolic function and LV afterload revealed no association with sex, implying that the real stroke work performed by LV myocardium might be the same for either sex. Given that healthy women appeared to have lower SBP compared with men, which might result in higher values of LVEF and more negative values of GLS for female myocardium to perform the same work but against a lower afterload. Hence, non-invasive myocardial work analysis might be a reliable tool to evaluate myocardial function independent of afterload conditions in either sex, beyond the standard assessment of LVEF and deformation.

In the meantime, our results confirmed but also extended previously published findings. We elucidated no influence of age, sex, BMI, HR, mitral annulus e′, LVMI, LV sizes or LVEF on GMW indices in young people, in accordance with the previous reports across the different age groups (10, 12, 13, 22). Interestingly, our study showed that SBP increased with age, whereas LV GLS decreases slightly with age in young people, which was in line with previous studies (22, 24). As the input variables for determining GMW indices, this inverse trend between SBP and LV GLS might explain the independence of GMW parameters from age. Likewise, our study demonstrated statistically significant relationship between both GWI and GCW and SBP and LV GLS, confirming GMW was more closely correlated with SBP than GLS. In addition, we further investigated that GWW and GWE were independently correlated with PSD, which describes the physiologic dyssynchrony of the longitudinal deformation. Specifically, higher PSD was positively related to GWW given dyssynchrony leads to less efficient work of LV myocardium and inhomogeneous distribution of glucose metabolism among myocardial segments (11, 25), which resulted in lower GWE. Chan et al. (26) newly reported significant increase of GWW in adult patients with non-ischemic dilated cardiomyopathy (DCM), associated with paradoxical deformation [post-systolic shortening (PSS) and early systolic lengthening (ESL)] of different myocardial segments (27). In our healthy population, in fact, increasing value of PSD was not an expression of LV dyssynchrony, which was in the normal range and correlated poorly with GMW parameters. Accounting for diastolic parameters, E/e′ ratio showed positively correlated with GWI and GCW, and E/A was positively correlated with GWE but negatively correlated with GWW on multivariable analysis. Of noted, LVMW analysis, integrating LV work performed during isovolumic relaxation, systolic ejection, and isovolumic contraction (from mitral valve closure to opening), provides complementary insights into LV contractility as well as segmental dyssynchrony. Therefore, GMW indices holds promise to further explore myocardial mechanics with clinical relevance in different disease entities, prior to conventional echocardiographic parameters such as LVEF or GLS.

In fact, the non-invasive LVMW analysis has been validated against invasive methods and demonstrated a strong correlation with myocardial oxygen consumption (5). Recent studies have demonstrated the incremental value of LVMW in diagnosis and prognostication compared with LVEF and myocardial strain in various diseased adult populations, such as a sensitive marker of early myocardial dysfunction in varied cardiac diseases including coronary artery disease (28), hypertension (29), hypertrophic cardiomyopathy (30, 31), dilated cardiomyopathy (26), heart failure with reduced or preserved ejection fraction, and a promising predictor of cardiac amyloidosis (32) or cardiac resynchronization therapy (CRT) response (33, 34). Nevertheless, the application of this new tool in young patients remains particularly understudied. Prior studies have shown that LV deformation is associated with outcomes in pediatric disease processes known to impair contractility, such as diabetes mellitus, chemotherapy cardiotoxicity, amyloidosis, Kawasaki disease, etc. However, as was previously mentioned, LV strain is often heavily influenced by loading conditions, thus failing to reflect true myocardial contractility. LVMW, indeed, adjusting myocardial deformation for LV pressure dynamics, could serve as a more robust metric in young patients for the serial assessment of LV performance under different hemodynamic conditions. In pediatric patients with dilated cardiomyopathy (DCM) (13 ± 4 years), Aly et al. (15) demonstrated strong correlation between the non-invasive GWE and exercise capacity, suggesting GWE as the main predictor of the maximal oxygen uptake (VO_2_ max). Of noted, as we had selected healthy young individuals in our study, the GMW markers were all within normal ranges. Therefore, additional validation should be done to optimize the use of LVMW analysis in young patients with various cardiovascular diseases.

### Limitations

Several limitations of our study should be acknowledged. First, LVMW is derived from 2D strain analysis, thus the accurate quantification of which would be influenced by the quality of ultrasound images. Nonetheless, the feasibility of the LVMW analysis was excellent with only 6% of healthy young volunteers excluded for poor image quality. Second, the non-invasive assessment of LV myocardial work is currently available on a single echocardiographic platform, and so far, cannot be evaluated using other software. Third, it was a prospective study, so the systemic arterial pressure was routinely obtained in the sitting position instead of left lateral decubitus, which might lead to discrepancy in blood pressure values and consequently affect the results of LVMW, as several studies reported posture affects the blood pressure (35, 36). Future studies are needed to explore the differences in blood pressure by body position in young people from different ethnics. Finally, we presented data from a single-center study in Chinese young people, so reference values might have to be adjusted in individuals of different descent. Also, larger-scale studies are needed for further evaluation of these new parameters to elucidate its clinical utility and prognostic implications.

## Conclusion

The echocardiographic reference values for non-invasive global myocardial work indices, independent of age, sex, BMI, HR, LVMI, LV sizes or LVEF in a large healthy young people, were reported to provide a foundation for incorporation into clinical practice. In addition, we revealed that GWI and GCW correlated more closely with SBP than GLS, while both GWW and GWE independently correlated with PSD. Our study suggests that LV GMW indices are reproducible and reliable, and may provide promising value for quantifying myocardial dysfunction in young patients with myocardial diseases.

## Data availability statement

The raw data supporting the conclusions of this article will be made available by the authors, without undue reservation.

## Ethics statement

The studies involving human participants were reviewed and approved by the Medical Ethics Committee of Shenzhen Hospital of Southern Medical University. Written informed consent from the participants’ legal guardian/next of kin was not required to participate in this study in accordance with the national legislation and the institutional requirements.

## Author contributions

XL and YZ conceptualized and designed the study, and coordinated and supervised the data collection. NZ, PL, XX, YC, DW, LM, and YM participated in the data collection. JS and QG took responsibility for the integrity of the data and the accuracy of the data analysis. XL screened the literature and wrote the first draft of the manuscript. YZ directed all phases of the study and revised the final manuscript. All authors reviewed and approved the manuscript as submitted and agreed to be accountable for all respects of the work.

## Abbreviations

2D: two-dimensional
STE: speckle-tracking echocar-diography
LV: left ventricle
LVEF: left ventricular ejection fraction
MW: myocardial work
GLS: global longitudinal strain
GMW: global myocardial work
SBP: systolic blood pressure
DBP: diastolic blood pressure
BMI: body mass index
BSA: body surface area
HR: heart rate
LVMI: left ventricular mass index
E: peak early transmitral flow velocity during diastole
A: peak late transmitral flow velocity during diastole
e′: early diastolic septal mitral annular velocity
PSD: longitudinal peak strain dispersion
GWI: global work index
GCW: global constructive work
GWW: global wasted work
GWE: global work efficiency
ICCs: Intra-class correlation coefficients.
